# Sex difference in circulating PCSK9 and its clinical implications

**DOI:** 10.3389/fphar.2022.953845

**Published:** 2022-09-07

**Authors:** Fang Jia, Si-Fan Fei, De-Bing Tong, Cong Xue, Jian-Jun Li

**Affiliations:** ^1^ Department of Cardiology, The Third Affiliated Hospital of Soochow University, Changzhou, China; ^2^ Cardio-Metabolic Center, Fu Wai Hospital, Chinese Academy of Medical Sciences, Peking Union Medical College, Beijing, China

**Keywords:** proprotein convertase subtilisin kexin type 9, sex difference, estrogen, coronary artery disease, PCSK9 inhibitor

## Abstract

Proprotein convertase subtilisin kexin type 9 (PCSK9) is a proprotein convertase that increases plasma low-density lipoprotein cholesterol (LDL-C) levels by triggering the degradation of LDL receptors (LDLRs). Beyond the regulation of circulating LDL-C, PCSK9 also has direct atherosclerotic effects on the vascular wall and is associated with coronary plaque inflammation. Interestingly, emerging data show that women have higher circulating PCSK9 concentrations than men, suggesting that the potential roles of PCSK9 may have different impacts according to sex. In this review, we summarize the studies concerning sex difference in circulating levels of PCSK9. In addition, we report on the sex differences in the relations of elevated circulating PCSK9 levels to the severity and prognosis of coronary artery disease, the incidence of type 2 diabetes mellitus, and neurological damage after cardiac arrest and liver injury, as well as inflammatory biomarkers and high-density lipoprotein cholesterol (HDL-C). Moreover, sex difference in the clinical efficacy of PCSK9 inhibitors application are reviewed. Finally, the underlying mechanisms of sex difference in circulating PCSK9 concentrations and the clinical implications are also discussed.

## Introduction

Major differences exist between men and women in the epidemiology, pathophysiology, and outcomes of cardiovascular diseases (CVDs) (Group et al., 2016). Significant variations in the prevention, clinical manifestations and treatment effects of CVDs according to sex persist worldwide, despite improvements in diagnostic and therapeutic interventions. Compared to men, women with acute coronary syndrome (ACS) are more likely to present with a range of atypical symptoms, including dyspnea, fatigue, and dizziness or weakness, instead of the classic symptoms of chest pain (An et al., 2019). In addition, women are characterized by a higher burden of cardiometabolic risk factors (Gerdts and Regitz-Zagrosek, 2019), a higher prevalence of nonobstructive coronary artery disease (CAD) on angiography (Bairey Merz et al., 2006; DeFilippis et al., 2020), and a higher prevalence of coronary microvascular dysfunction compared to men (Waheed et al., 2020). The nonspecific chest pain and nonobstructive CAD often observed in women do not confer a lower risk for recurrent acute myocardial infarction (AMI) and mortality, and the prognosis in these women is not benign (Robinson et al., 2008; Kenkre et al., 2017). Moreover, women with established atherosclerotic cardiovascular disease (ASCVD) are less likely to use specific guideline-directed medications for secondary prevention than men (Xia et al., 2020). Several studies have demonstrated less favorable outcomes in women with ACS than in men and worse all-cause mortality following primary percutaneous coronary intervention (PCI) after ST-elevation myocardial infarction (STEMI) (Pagidipati and Peterson, 2016; Rathod et al., 2021).

Sex differences in CVD are due to differences in gene expression and the regulation of sex hormones that lead to differences in various cardiovascular functions, for example, in nitric oxide (NO) signaling, myocardial remodeling under stress, glucose regulation and lipid metabolism (Group et al., 2016). Preclinical evidence on sex differences and pathophysiological clarification may contribute to the development of sex-specific therapeutic strategies. Deciphering sex-specific differences in biomarkers may improve our understanding of the associated biological mechanisms. The circulating level of proprotein convertase subtilisin kexin type 9 (PCSK9), which is associated with the severity and outcomes of patients with CAD, has been described as a new risk marker of CAD (Bae et al., 2018; Panahi et al., 2019; Peng et al., 2020; Kajingulu et al., 2022). An animal model evaluating the sex-specific distribution of low-density lipoprotein receptors (LDLRs) shows that the absence of PCSK9 results in a sex- and tissue-specific subcellular distribution of LDLRs, suggesting that PCSK9 and estrogen may act as molecular regulators of cholesterol homeostasis, and PCSK9 inhibitors may have different effects in women than in men (Roubtsova et al., 2015). The range of circulating PCSK9 concentrations is broad and differs between sexes. The current review focuses on sex difference in circulating PCSK9 levels and the potential mechanisms and clinical implications for these observed sex-based differences.

## Methods

This systematic review examined sex difference in circulating PCSK9 levels and the underlying mechanisms and clinical implications of these sex differences. The selected studies included cross-sectional, case-control and prospective studies. There were no language or time restrictions for eligible studies. The PubMed electronic database was used. The following search terms were used: “proprotein convertase subtilisin kexin type 9” OR “PCSK9” AND “sex difference” AND “estrogen” OR “estradiol” AND “coronary artery diseases” OR “type 2 diabetes mellitus” OR “neurological damage after cardiac arrest” OR “liver injury” OR “inflammatory biomarkers” OR “HDL-C” OR “PCSK9 inhibitor”.

### Sex difference in circulating PCSK9 concentrations

Increasing evidence shows that PCSK9 is a well-known therapeutic target in the prevention and treatment of atherosclerosis. PCSK9 is an important enzyme in cholesterol metabolism that regulates serum low-density lipoprotein cholesterol (LDL-C) levels through the degradation of LDLRs. PCSK9 inhibitors could lead to significant LDL-C reductions in high-risk patients, and they have shown a favorable safety profile in recent clinical trials (Robinson et al., 2015; Sabatine et al., 2017). Beyond the regulation of circulating LDL-C levels, PCSK9 also has direct atherogenic effects on the vascular wall and is associated with coronary plaque inflammation (Gencer et al., 2016; Tang et al., 2017; Liu and Frostegard, 2018). PCSK9 can interfere with the underlying molecular mechanisms in atherosclerosis, from endothelial dysfunction to smooth muscle cell migration and the activation of inflammatory pathways (Ding et al., 2015; Ferri et al., 2016; Liu et al., 2020). All these findings consistently indicate that PCSK9 plays a significant role in every step of the development and progression of coronary plaque.

Although the potential role of PCSK9 still remains to be elucidated, many studies in humans have shown that there is a significant sex difference in circulating levels of PCSK9. PCSK9 levels have been measured in many studies, which have shown a broad range of concentrations. The PCSK9 level was significantly higher in women than in men (331 ± 105 ng/ml vs. 290 ± 109 ng/ml) in the IMPROVE cohort study, a large-scale multicenter study encompassing several European countries with the centralized measurement of PCSK9 (Ferri et al., 2020). The effect of sex on PCSK9 concentrations was also observed in a population-based prospective study conducted among 4,205 Chinese participants with prediabetes (average age 56.1 ± 7.5 years), as circulating PCSK9 levels were higher in females than in males (289.62 ± 98.80 ng/ml vs. 277.50 ± 97.57 ng/ml, *p* < 0.001) (Shi et al., 2020). Although the clinical implications of elevated circulating PCSK9 levels are still unclear in AMI patients, circulating PCSK9 are higher in women than in men not only for all admitted AMI patients, but also for patients with STEMI (Zhang et al., 2019). Many studies concerning PCSK9 levels have been conducted in elderly people with high ASCVD risk or established ASCVD; however, it is notable that sex difference in circulating levels of PCSK9 also exists in young people (Levenson et al., 2017). Levenson’s study showed that PCSK9 levels in young men were lower than those in young women, which was consistent with findings in elderly individuals. As listed in Table 1, these studies have demonstrated similar findings that PCSK9 levels are higher in women than in men.

**TABLE 1:** Demographic features and PCSK9 levels in the observational studies comparing circulating PCSK9 levels between women and men.

Author, year	Study design	Number of participants (women vs. men)	Location	Age (y)	PCSK9 levels (women vs. men)	Hr (95%CI)
Ferri et al. (2020)	Prospective	1929 vs. 1774	Five European countries: Finland, Sweden, the Netherlands, France, and Italy	54 to 79	331 ± 105 vs. 290 ± 109 ng/ml	*p* < 0.0001
Shi et al. (2020)	Prospective	2,817 vs. 1,388	China	56.1 ± 7.5	289.62 ± 98.80 ng/ml vs. 277.50 ± 97.57 ng/ml	*p* < 0.001
Zhang et al. (2019)	Prospective	61 vs. 220	China	Males: 58.53 ± 12.83; Females: 68.64 ± 8.97	325.1 (247.5–445.3) vs. 273.6 (215.6–366.8) ng/ml	*p* = 0.0136
Levenson et al. (2017)	Cross-sectional analysis	167 vs. 119	United States	15 to 26	—	*p* = 0.03
Simeone et al. (2021)	Observational	67 vs. 99	Italy	68	308 (251–394) vs. 267 (231–340) ng/ml	*p* = 0.005
Jeenduang. (2019)	Cross-sectional analysis	284 vs. 152	Thailand	52.50 ± 13.56	87.38 ± 26.49 vs. 79.15 ± 25.51 ng/ml	*p* = 0.002

### Potential mechanisms

Circulating PCSK9 appears to be produced mainly by the liver, and its expression is regulated by numerous factors, such as the diurnal rhythm (Persson et al., 2010), insulin (Levenson et al., 2017), resistin (Macchi et al., 2020), thyroid hormone (Yildirim et al., 2021), diet (Krysa et al., 2017), exercise (Sponder et al., 2017), and various cholesterol-lowering drugs (Sahebkar et al., 2015). It seems that sex could modify the effects of extrinsic and intrinsic factors on the PCSK9 concentration. Even though a wealth of data exists regarding sex differences in CVD and their underlying risk factors, a comprehensive understanding is still lacking. Genetic mechanisms, based on the differences in sex chromosomes, sex hormones and their receptors, are speculated to play a major role. The effect of estrogens on lipid homeostasis and circulating levels of PCSK9 has received increasing attention. Postmenopausal women have higher PCSK9 concentrations than premenopausal women, which may be related to the decrease in estrogen during menopause (Ghosh et al., 2015). In men, no correlation has been found between serum testosterone and plasma PCSK9 levels, and testosterone replacement therapy does not have an effect on plasma PCSK9 levels (Ooi et al., 2015). In Ghosh M’s study, females over 50 years of age (330 ng/ml) had higher PCSK9 levels than those below 50 years of age (276 ng/ml; *p* < 0.05), whereas the two groups of male participants had similar PCSK9 levels (279 vs. 270 ng/ml; *p* > 0.05) (Ghosh et al., 2015).

The regulating effects of estrogen on PCSK9 expression noted in recent studies are as follows: 1) PCSK9 levels increase in women after menopause, along with the sharp reduction in estrogen that occurs during this time period; however, PCSK9 levels do not increase in men during this time period (Ghosh et al., 2015). 2) PCSK9 levels change throughout the menstrual cycle, and an inverse relation exists between PCSK9 and estradiol in premenopausal women. The estradiol level is the highest at ovulation, while the PCSK9 level, on average, is 235 ng/ml in this phase, which is lower than that in the luteal and follicular phases (Ghosh et al., 2015). 3) The inverse relationship between PCSK9 and estradiol is also found in women preparing for *in vitro* fertilization. *In vitro* fertilization involves 2 treatment phases: Extreme suppression and strong stimulation of the endogenous estrogen levels. A comparison of PCSK9 levels in these two phases shows that PCSK9 levels are significantly reduced by 14% after the stimulation of estrogen synthesis, which indicates that high levels of endogenous estrogens reduce circulating PCSK9 levels (Persson et al., 2012).

**FIGURE 1:**
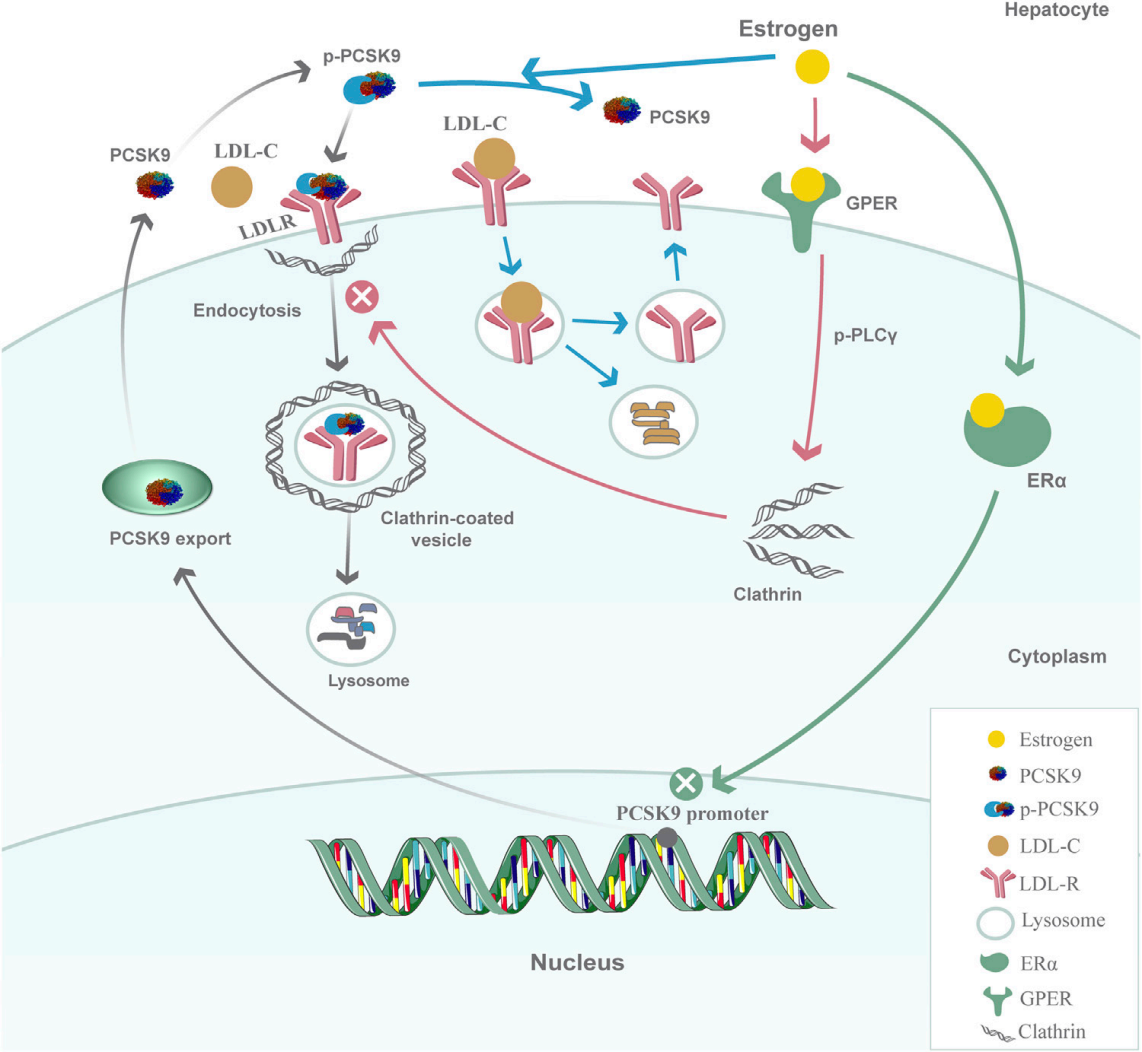
Model for the regulation of PCSK9 by estrogen in hepatic cells. At the transcriptional level, estrogen inhibits PCSK9 proximal promoter activities and decreases PCSK9 mRNA expression through the ERα-mediated pathway; at the posttranscriptional level, the activation of GPER in the membrane by estrogen rapidly initiates the phosphorylation of PLCγ and then alters clathrin distribution or influences the clathrin trafficking pathway to affect the internalization of the PCSK9-LDLR complex. Another possible mechanism is that estrogen could affect the PCSK9-LDLR interaction by inhibiting the phosphorylation of PCSK9 to protect LDLRs from degradation.

A deep understanding of the mechanisms by which PCSK9 is regulated by estrogen could be beneficial for clarifying the sex difference in PCSK9 concentrations and its effects on atherosclerotic disease progression and cardiovascular outcomes. Estrogen is speculated to affect PCSK9 expression and function through a transcriptional and posttranscriptional mechanism (Figure 1). At the transcriptional level, estradiol and phytoestrogens suppress PCSK9 proximal promoter activities in human L02 hepatocytes through an estrogen receptor α (ERα)-mediated pathway (Jing et al., 2019). G-protein estrogen receptor (GPER), a well-recognized receptor activated by estrogens, has been increasingly found to mediate the physiological effects of estrogen. GPER activation can decrease PCSK9 mRNA and protein levels in human hepatic cell lines, thus suggesting its influence on the expression of PCSK9 (Hussain et al., 2015). *In vitro* studies have also found that estrogens could affect the PCSK9 functional state through a posttranscriptional mechanism. One of the possible mechanisms for the posttranscriptional inhibiting effect is that estradiol could block PCSK9 internalization in hepatic cells. The activation of GPER in the membrane by estradiol rapidly initiates the phosphorylation of phospholipase C-γ (PLC-γ), which in turn alters clathrin distribution or influences the clathrin trafficking pathway to affect the internalization of the PCSK9-LDLR complex in HepG2 cells (Fu et al., 2019). The other mechanism is that estradiol causes a functional switch in the PCSK9-LDLR interaction through phosphorylation. Starr’s results showed a dynamic role of PCSK9 under estradiol conditioning at the protein level because estradiol treatment of HuH7 cells resulted in decreased phosphorylation of secreted PCSK9, which was associated with the protection of LDLRs (Starr et al., 2015). Taken together, these findings show that the regulation of estrogen and its receptors on PCSK9 inhibits PCSK9 function by suppressing PCSK9 proximal promoter activities, impairing PCSK9-LDLR complex internalization, and dephosphorylating secreted PCSK9 in hepatic cells, preventing it from escorting LDLRs to lysosomes for degradation. The upregulation of LDLRs by estradiol, at least in part, is likely to occur through PCSK9 downregulation, which in turn would lead to decreased circulating levels of LDL-C. Investigations into other possible mechanisms for the regulation of PCSK9 under the action of estrogen would be of great interest.

### Clinical implications of sex difference in circulating PCSK9

Although men and women differ in many aspects, including genetic mechanisms and epigenetic mechanisms, estrogen is a recognized regulator that is significantly associated with the discrepancy in circulating PCSK9 levels between men and women. The relations between elevated circulating PCSK9 levels and clinical conditions such as the severity and prognosis of CAD, the incidence of type 2 diabetes mellitus (T2DM), neurological damage after cardiac arrest, liver injury as well as inflammatory biomarkers and HDL-C are complex; moreover, the clinical implications of sex difference in PCSK9 levels need to be clarified.

#### Severity and prognosis of coronary artery disease

It is clear that PCSK9 contributes to every step of the molecular pathway of atherosclerosis. Recent studies have revealed that PCSK9 has various effects on the progression of atherosclerosis, including inflammation, foam cell formation, endothelial cell apoptosis, smooth muscle cell phenotypic switching, and platelet activation (Yurtseven et al., 2020; Puteri et al., 2022). Many clinical studies have tested the relation between circulating levels of PCSK9 and the presence and severity of CAD. The prevalence of coronary atherosclerotic lesions and the Gensini score increased as the circulating PCSK9 levels increased in patients with familial hypercholesterolemia (Cao et al., 2018a). In patients with CAD, circulating PCSK9 levels were found to be positively associated with severity scores (SYNTAX, Gensini, and Jeopardy) (Li et al., 2015a; Li et al., 2015b). In addition, PCSK9 levels were positively associated with the severity of coronary artery lesions independent of LDL-C concentrations in patients hospitalized for ACS (Cariou et al., 2017). However, a cross-sectional study in China explored associations of circulating PCSK9 levels and lipid parameters (LDL-C, non-HDL-C, apolipoprotein B, lipoprotein (a), etc.) with coronary artery lesion severity in non-lipid-lowering-drug-treated patients undergoing their first coronary angiography, and a positive association with the Gensini score was found in men but not in women (Li et al., 2017). A larger sample may be needed to confirm the lack of an association between PCSK9 levels and coronary artery lesion severity in women, as many CAD studies have included mostly men. Furthermore, the Gensini score has always been used as a surrogate marker of coronary artery lesion severity; however, it might not be fully representative of the anatomical and morphological features of severe coronary lesions.

Invasive and pathological evidence has suggested sex-specific differences in the pattern of compositional plaque progression: Men are more likely to have plaque rupture and occlusion that is associated with sudden onset symptoms than women, and women can present with plaque rupture as well as plaque erosion and an indolent course of anginal symptoms (Yahagi et al., 2015; Chandrasekhar and Mehran, 2016). Women suffer from higher rates of coronary microvascular dysfunction, possibly because they are particularly predisposed to mental stress and neuroendocrine dysfunction (Safdar et al., 2018). A positive association between circulating PCSK9 levels and the fraction of plaque consisting of necrotic core tissue (an index of plaque vulnerability) was documented by intravascular ultrasound (IVUS) in patients with stable CAD or ACS (Cheng et al., 2016). In addition, PCSK9 was demonstrated to be associated with aggravated microvascular obstruction. PCSK9 was found to aggravate microvascular obstruction and promote myocardial infarction (MI) expansion post-MI in MI mouse models, and a PCSK9 inhibitor weakened the enhanced platelet aggregation and ameliorated microvascular obstruction (Qi et al., 2021). The association between elevated circulating PCSK9 levels and coronary plaque morphology and coronary microvascular dysfunction may affect sex differences in the composition, progression and clinical presentation of coronary plaque.

In addition to the associations between PCSK9 levels and coronary artery lesion severity in women, knowledge of the relation of PCSK9 to CAD outcomes may also require further, more specific exploration. Although sex differences in CAD outcomes are not consistent among all reports, many studies have shown that female patients do not have more favorable outcomes than male patients; moreover, several studies have demonstrated an even worse prognosis in female patients than male patients. Women with stable angina and nonobstructive CAD were found to be 3 times more likely to experience a cardiac event within the first year of cardiac catheterization than men (Sedlak et al., 2013). Compared to men, women have a higher risk of death and adverse outcomes after primary PCI for STEMI (Kosmidou et al., 2017). A meta-analysis of the prognosis of young women compared with that of men demonstrated that young women with ACS may have a variety of nontraditional risk factors, and the in-hospital, short-term and long-term mortality rates of these female patients were higher than those of male patients (Ma et al., 2017).

The circulating PCSK9 level is independently predictive of major adverse cardiovascular events (MACEs) in patients with stable CAD (Li et al., 2015b). PCSK9 was shown to be independently associated with an increased number of ischemic MACEs in ACS patients undergoing PCI at the 1-year follow-up: The hazard ratio for upper vs. lower PCSK9-level tertiles was 2.62 (*p* = 0.01) (Navarese et al., 2017). A prospective, observational cohort study of 1,225 untreated patients with stable CAD showed that the group with high PCSK9 levels (≥234.52 ng/ml) had a significantly higher risk of MACEs than the group with low PCSK9 levels (<234.52 ng/ml) during a median of 3.3 years of follow-up, while patients in the group with high PCSK9 levels were more likely to be female (Peng et al., 2020). The same results were observed in another prospective study including 504 consecutive patients with stable CAD, the majority of whom were receiving statin treatment (Werner et al., 2014). Patients with higher PCSK9 levels had more primary adverse events, which included cardiovascular death and unplanned cardiovascular hospitalization, and women accounted for a higher proportion of these patients. An investigation of serum PCSK9 levels in patients undergoing PCI also demonstrated that a higher serum PCSK9 level was independently associated with a higher rate of MACEs and all-cause death compared with a lower serum PCSK9 level, and the proportion of women was higher in the group with high PCSK9 levels (Choi et al., 2020). The sex differences in the outcomes of CAD may at least be partially related to the sex difference in circulating PCSK9 levels, which is related to specific coronary plaque features and coronary microvascular dysfunction. Therefore, a better understanding of the sex differences in the pathogenesis of coronary atherosclerosis and the role of PCSK9 could lead to the selection of appropriate preventive measures to improve both the quality of life and clinical outcomes in women.

#### Incidence of T2DM

The findings from the available clinical study on PCSK9 and T2DM suggest a trend toward a positive association between circulating PCSK9 levels and the incidence of T2DM in renal transplant recipients (Eisenga et al., 2017). Moreover, insulin and glycemic parameters of diabetes mellitus (DM), such as the homeostasis model assessment of insulin resistance (HOMA-IR) and glycated hemoglobin (HbA1c) level, are positively correlated with the circulating PCSK9 concentration (Peng et al., 2020; Hamamura et al., 2021). Nevertheless, these findings are not consistent among all reports (Ramin-Mangata et al., 2020). A population-based prospective study in China showed that the positive association between circulating PCSK9 levels and the risk of incident T2DM was found only in female participants with prediabetes; conversely, no significant association was observed among male prediabetic participants, which revealed the sex discrepancy in the relation between elevated circulating PCSK9 levels and the incidence of T2DM (Shi et al., 2020).

We suggest that gonadal hormones may be an important confounding factor for the relationship between circulating PCSK9 levels and the incidence of T2DM. Circulating PCSK9 levels change in women depending on their reproductive stage of life, and serum estrogen is inversely correlated with circulating PCSK9 levels, while in men, serum testosterone is not correlated with circulating PCSK9 levels (Ooi et al., 2015; Mauvais-Jarvis, 2018). In women, an early menopausal age (before the age of 45 years) is associated with an increased risk of diabetes compared to an older menopausal age (Shen et al., 2017), and the rapid and severe estrogen deficiency following surgical ovariectomy is also accompanied by an increased diabetes risk (Pandeya et al., 2018). The mechanisms for facilitating glucose homeostasis in women before menopause could be, at least in part, due to the beneficial effect of a physiological window of circulating estrogens. Sex hormones play a role in these sex differences in glucose homeostasis, prediabetic syndromes and diabetes and might affect the relation between circulating PCSK9 levels and the incidence of T2DM.

#### Neurological damage after cardiac arrest

Cardiac arrest causes significant morbidity and mortality, and women have been found to have worse outcomes despite improvements in prehospital and hospital care. Women were associated with a lower likelihood of good neurological outcomes at discharge and the 6-month follow-up in a multinational retrospective registry of patients who suffered out-of-hospital cardiac arrest (Vogelsong et al., 2021). However, data from the Cardiac Arrest Registry to Enhance Survival (CARES) indicate that men have lower rates of favorable neurological survival than women (Kotini-Shah et al., 2021). Several large Asian studies have found no sex differences in the rates of neurological survival between men and women (Ng et al., 2016; Goto et al., 2019). The reasons for these differences are complex and involve the pathophysiological features of the disease and its comorbidities, resuscitative care protocols, and the response to treatment (Jarman et al., 2019).

An extensive investigation of PCSK9 revealed its novel potential functions, including the regulation of neuronal development, apoptosis and differentiation, but the precise role of PCSK9 in brain physiology remained unclear (Mannarino et al., 2018). Moreover, high circulating PCSK9 levels were associated with unfavorable neurological function after resuscitation from out-of-hospital cardiac arrest (Merrelaar et al., 2020). The increase in PCSK9 levels was most likely caused by the inflammatory response and organ dysfunction after cardiopulmonary resuscitation (CPR). The favorable neurological outcomes in patients with low circulating PCSK9 levels may be attributed to a more rapid detoxification of bacterial lipids via higher LDL-R expression and a reduced inflammatory response. However, the sample size of the study was limited, and only 61 men and 18 women were enrolled. Thus, analyses in larger populations are needed to verify the role of PCSK9 in neurological outcomes after resuscitation and to explore the differences in neurological outcomes between women and men with different circulating PCSK9 levels.

#### Liver injury

PCSK9 has recently been shown to influence inflammatory responses in the liver. Circulating PCSK9 levels were associated with steatosis severity in patients who underwent liver biopsy for suspected nonalcoholic steatohepatitis (Ruscica et al., 2016). The mean PCSK9 levels in patients with end-stage liver disease and mixed disease etiology were much lower than those in healthy controls (Schlegel et al., 2017). Although PCSK9 expression differs in different stages of liver cirrhosis and different etiologies of liver injury, there is increasing evidence that PCSK9 contributes to the pathogenesis of nonalcoholic fatty liver disease (NAFLD). Mice that overexpressed PCSK9 with a high-fat diet had increased hepatic steatosis, macrophage infiltration and fibrosis scores (Grimaudo et al., 2021).

PCSK9 inhibition seems to exert a protective effect against hepatic damage in NAFLD. A PCSK9 loss-of-function variant in humans was protective against liver steatosis and fibrosis in individuals with NAFLD (Grimaudo et al., 2021). The genetic deletion of PCSK9 improved liver inflammation and fibrosis in bile duct-ligated mice and reduced liver function markers such as alanine transaminase (ALT) and aspartate transaminase (AST) levels, suggesting that PCSK9 inhibition can rescue liver inflammation and hepatocyte injury (Zou et al., 2020). PCSK9-targeted therapies could be a potential therapeutic approach to ameliorate NAFLD, which is closely related to atherosclerotic disease and cardiovascular risk factors. However, one study including 202 hyperlipidemia patients found that there was a mean increase of 5.8 mg/dl and 6.2 mg/dl from baseline in ALT and AST levels, respectively, in patients who were taking PCSK9 inhibitors compared to those who were not taking PCSK9 inhibitors (Zafar et al., 2020). This study had a small sample size and no long-term follow-up, and no alcohol use or detailed liver comorbidities, such as NAFLD, were documented. To obtain a better understanding of the relation between plasma PCSK9 levels and NAFLD or other chronic liver diseases, more intensive studies are needed to estimate sex differences in the impact of PCSK9 inhibition in patients with metabolic liver diseases. In Ruscica M’s study, circulating PCSK9 levels were not significantly associated with female sex in a multivariate analysis, and it was hypothesized that the induction of PCSK9 in NAFLD may overcome its regulation by sex hormones (Ruscica et al., 2016). This hypothesis also needs to be evaluated in larger studies.

#### Inflammatory biomarkers

The difference in vascular inflammation between men and women has recently become the focus of many studies (Shabbir et al., 2021). The proinflammatory role of PCSK9 in atherosclerosis has been supported by many studies. Higher levels of plasma PCSK9 were independently associated with inflammatory markers such as the white blood cell count (WBCC), fibrinogen levels, and high sensitivity C-reactive protein (hs-CRP) levels in patients with ACS and CAD (Li et al., 2015a). Moreover, PCSK9 has been found to enhance the production of proinflammatory cytokines; for example, the TLR4/NF-κB signaling pathway could be stimulated to mediate the PCSK9-induced increase in the inflammatory response (Tang et al., 2017). The effects of PCSK9 on CAD were found to be mediated partly by inflammation in addition to lipid metabolism. However, in the analysis performed based on sex, the relation between PCSK9 levels and the WBCC remained significant only in men (Li et al., 2014). Moreover, there was no differential effect of PCSK9 monoclonal antibody therapy on plasma hs-CRP concentrations, and no statistically significant relation between sex and hs-CRP changes was observed in Ye-Xuan Cao’s meta-analysis (Cao et al., 2018b). Thus, there might be a different link between PCSK9 levels and inflammatory biomarkers according to sex, but recent data are still limited by sample size. Further studies are necessary to identify sex differences in the relation between PCSK9 levels and inflammatory markers.

#### High-density lipoprotein cholesterol

HDL-C is inversely associated with CVD across a wide range of concentrations. Indeed, different HDL subpopulations may have different functional properties since HDL particles are heterogeneous in size and biochemical composition. Numerous studies have shown that small- and medium-sized HDL particles are inversely related to cardiovascular risk (McGarrah et al., 2016; Duparc et al., 2020). On average, women have higher HDL-C levels than men, and the corresponding concentration of HDL-C associated with the lowest all-cause mortality for women (2.4 mmol/L) is also higher than that for men (1.9 mmol/L) (Madsen et al., 2017). PCSK9 monoclonal antibodies have been reported to cause not only a moderate increase in HDL-C levels but also an increase in medium-sized HDL particles (Zhang et al., 2015; Ingueneau et al., 2020). Plasma PCSK9 levels are positively correlated with small HDL particles; however, the relation of PCSK9 levels to HDL-C is complicated because the interaction between HDL particles and PCSK9 alters PCSK9 functionality (Burnap et al., 2021). HDL particles have been shown to promote the multimerization of PCSK9 and act as facilitators of PCSK9-driven LDLR degradation. In addition, PCSK9 binds to LDL particles, and LDL particles can inhibit the effects of PCSK9 on LDLRs (Kosenko et al., 2013); thus, lipoproteins dynamically alter PCSK9 function. Sex differences exist in the interaction between PCSK9 levels and HDL particles because PCSK9 is significantly enriched in HDL particles isolated from females (Burnap et al., 2021). Further exploration is needed to determine sex differences in PCSK9 activity and whether the role of sex in the HDL-PCSK9 relation might impact the efficacy of PCSK9 inhibition.

### Sex difference in the clinical efficacy of PCSK9 inhibitor application

Despite the overwhelming evidence of cardiovascular benefits from trials with lipid-lowering medications, there is evidence that women are often undertreated in clinical practice (Bittner et al., 2015; Nanna et al., 2019). Moreover, women have been shown to experience more statin-associated side effects than men, and they may discontinue therapy because of their higher susceptibility to statin-associated adverse events (Karalis et al., 2016). However, in a recent analysis including a US nationwide sample of Medicare beneficiaries who were hospitalized for MI and had very high ASCVD risk, women were more likely to initiate treatment with PCSK9 inhibitors (Colvin et al., 2021). Therefore, in light of the higher circulating PCSK9 levels in women, it is important to establish whether treatment with PCSK9 inhibitors, which produce substantial reductions in LDL-C, would confer consistent cardiovascular benefits in both sexes.

One concern related to PCSK9 inhibitors is whether they may increase the risk of impaired glucose metabolism and the development of new-onset diabetes; however, recent clinical trial data for PCSK9 monoclonal antibodies suggest that the application of alirocumab and evolocumab in the treatment of atherosclerotic disease does not increase the risk of new-onset diabetes or worsen glycemia. In addition, the inhibition of PCSK9 by polydatin, a natural antidiabetic product, could modify glucose metabolism disorders and thereby ameliorate diabetic complications by increasing the expression of liver glucokinase, a key enzyme in glucose metabolism (Wang et al., 2016). Nonetheless, it remains unclear whether treatment with PCSK9 inhibitors can exert a positive effect to alleviate glycemic parameters and whether there are sex differences in the effects of PCSK9 inhibitors on glucose metabolism in patients with DM.

In the FOURIER trial, the LDL-C reduction with evolocumab at 4 weeks was nominally greater in men than women, but the relative risk reductions in endpoint analysis were similar in women and men. The large size of the FOURIER trial population provides a robust evidence base that the absolute risk reductions of adverse events with evolocumab application were similar in both men and women, and no important safety issues were observed in either sex (Sever et al., 2021). Consistently, the preliminary results of the ODYSSEY OUTCOMES trial with alirocumab showed that the relative risk reductions for the primary composite endpoint were broadly similar in women and men (9 and 17%, respectively, P interaction = 0.35) (Schwartz et al., 2018). In conclusion, the benefits of PCSK9 monoclonal antibodies were found to be similar in both men and women with high ASCVD risk in recent studies.

The administration of PCSK9 monoclonal antibodies to patients with hyperlipidemia and CAD led to a 50–70% reduction in LDL-C levels (Sabatine et al., 2017; Sinnaeve et al., 2020). Small-molecule PCSK9-targeting agents have been found to be effective competitors for both PCSK9 monoclonal antibodies and siRNA (Salaheldin et al., 2022). P-21, considered the first oral small-molecule nanohepatic targeted anti-PCSK9/LDLR compound, seems to offer a more efficient, safer, and easier-to-administer treatment protocol. In a hypercholesterolemia mouse model, P-21 led to a reduction of more than 90% in LDL-C levels after 1 week of treatment, and toxicology studies in rats showed normal chemical biomarkers and normal histopathological findings, with no apparent toxic clinical signs (Salaheldin et al., 2022). The recent innovations in PCSK9 inhibitors have ushered in a new era in lipid-lowering therapy; however, more sex-specific subanalyses are necessary, and sex differences in their clinical efficacy should be estimated.

## Conclusion

Although the overall mortality in patients with CAD has dramatically declined over recent decades as a result of preventive strategies, the decline has been less significant for women (Benjamin et al., 2017). The physiological roles of PCSK9 need to be further investigated because its functions are far more than the regulation of plasma LDL-C levels. Circulating PCSK9 levels are higher in women than in men, and postmenopausal women have higher PCSK9 concentrations than premenopausal women. Estrogen could affect circulating PCSK9 concentrations, probably through transcriptional and posttranscriptional mechanisms. The sex differences in circulating PCSK9 levels call for clinical attention and may represent a pharmacological target for the prevention and treatment of CVDs in women. The potential of PCSK9 in the prediction of ASCVD risk and MACEs based on sex differences requires further prospective investigation. Thus, sex-specific subanalyses may be warranted, and information regarding hormonal status should be taken into account, especially for women. This information might be taken into consideration when defining individual risk for cardiovascular events and/or refining PCSK9-lowering treatments.
